# Analysis of risk variables for association with maxillary sinus mucosal thickenings: a cone-beam computed tomography-based retrospective study

**DOI:** 10.1007/s00276-023-03090-2

**Published:** 2023-02-08

**Authors:** Carolina Betin-Noriega, Samuel Enrique Urbano-del Valle, Clara Inés Saldarriaga-Naranjo, Jorge Luis Obando-Castillo, Sergio Iván Tobón-Arroyave

**Affiliations:** 1 Graduate Oral and Maxillofacial Surgery Program, Faculty of Dentistry, University of Antioquia, Medellín, Colombia; 2 Department of Radiology, CES University and RADEX 3D Specialized Radiology Center, Medellín, Colombia; 3 Department of Radiology, Cooperative University of Colombia, San Juan de Pasto, Colombia; 4 Laboratory of Immunodetection and Bioanalysis, Faculty of Dentistry, University of Antioquia, Calle 70 N° 52-21, Medellín, Colombia

**Keywords:** Cone-beam computed tomography, Diagnostic imaging, Maxillary sinus, Risk assessment, Etiology

## Abstract

**Purpose:**

This study aimed to determine which patient-related, anatomical, pathologic, or iatrogenic variables may be directly associated with and which may have a modifying effect on the generation of maxillary sinus (MS) mucosal thickenings.

**Methods:**

A total of 278 cone-beam computed tomography (CBCT) scans obtained from 114 males and 164 females were evaluated. The protocol included the assessment of 21 candidate variables, of which 18 were bilateral and 3 were unique. The relationship among the study variables and the mucosal thickenings were examined individually and adjusted for confounding using univariate and multivariate binary logistic regression models.

**Results:**

The prevalence of mucosal thickenings was 71.20% at patient level and 53.40% at sinus level. The ostium height > 28.15 mm, the infundibulum length ≤ 9.55 mm, the infundibulum width ≤ 0.50 mm, along the occurrence of periapical lesions and slight-to-severe periodontal bone loss acted as strong/independent risk variables for MS mucosal thickenings. Confounding and interaction relationships between MS height and depth, and between the alveolar process type and the presence of foreign materials with respect to age stratum > 47.50 years might be also associated with the mucosal thickenings.

**Conclusions:**

While increased ostium height, decreased infundibulum length/width, the presence of periapical lesions and periodontal involvement might be the foremost indicator variables for MS mucosal thickenings, there are synergistic relationships among the increased sinus height and depth as well as aging regarding atrophic/partially atrophic alveolar process status and the presence of foreign materials that may be also associated with a greater proportion of these mucosal abnormalities.

## Introduction

Maxillary sinuses (MS) are a pair of pyramid-shaped pneumatic cavities located within maxillary bone that communicate with the nasal cavity through the ostium [20]. They are lined by thin respiratory mucous membrane composed of pseudostratified cylindrical ciliated epithelium named sinus mucosa that adheres firmly to the periosteum and, under normal conditions, has approximately 0.8–1 mm thick [6, 20]. Nevertheless, there is not yet a general consensus that allows establishing when the sinus mucosa is altered, as while it has been proposed that it should not be visible on a radiograph [20] and that any thickening should be considered anomalous [13], some authors have defined a more precise limit of mucosal thickening above which it would be correct to diagnose a sinus pathological condition [43], so that increasing evidence suggests that thickenings ≥ 2 mm should be considered indicative of a sinus disorder [13, 39, 46]. As such, the alterations in the sinus mucosa may appear either as uniform thickenings and hypertrophic areas, or as polypoids, solid, or cystic masses [9] which can obstruct the ostium.

Several authors mention the mucosal thickenings as the most frequent finding in MS, with a prevalence ranging from 34.6 to 68.8% [3, 8, 9, 23, 31, 39, 42, 43] at patient level, while at sinus level, the prevalence rates have been estimated between 29.4 and 66%. [3, 42, 46]. This broad range of variability could be attributable not only to differences in various methodological aspects used in the investigations [41], such as selection of cases, sample size, imaging techniques, variables analyzed, and statistical methods, but also to discrepancies in the diagnostic criteria used in the evaluation [43], which might damp interpretation of the results. While the radiographic detection of alterations in sinus mucosa per se does not allow establishing a differential diagnosis between acute and chronic sinus disease [9], mucosal disorders constitute a finding of interest because they may represent a sinus pathology of inflammatory, allergic, traumatic, neoplastic, or congenital origin [23, 38, 49], as well as an indicator of potential complications for surgical procedures involving MS [29].

Most of the available studies assessing the etiology of MS mucosa alterations have only focused in individual risk indicators as explanatory variables. From these studies, it has been concluded that certain potential predictors, including demographic [17, 23, 51, 52], anatomical [13, 17, 26, 42], iatrogenic [1, 32], and pathologic [3, 9, 14, 15, 20, 32, 38, 49] features may be associated with the presence of these abnormalities, but actually few researches have analyzed the combined effect of multiple variables [29], so that there is only sparse information about those variables that might influence this association as potential confounders or on their biological interaction effects [18, 27, 29, 41].

Bearing in mind not only that sinus mucosa thickenings have a multicausal etiology, but also that cone-beam computed tomography (CBCT) constitutes a complete and precise method for the diagnosis of sinus alterations due to its high resolution and the possibility of visualizing both hard and soft tissues [9], this study aimed to determine which patient-related, anatomical, pathologic, or iatrogenic variables may be strongly and independently associated and which may have a modifying effect on the generation of these mucosal abnormalities.

## Materials and methods

### Study design and inclusion/exclusion criteria

This cross-sectional analytic study was performed in compliance with the ethical guidelines of the Helsinki Declaration, on CBCT scans of patients attending a private imaging specialized center (RADEX 3D Specialized Radiology Center) in Medellín, Colombia. The manuscript was prepared in compliance with the *Strengthening the Reporting of Observational Studies in Epidemiology* (STROBE) statement for cohort, case–control, and cross-sectional studies (https://www.strobe-statement.org). The sample size was estimated based on the total number of patients referred for complete skull, bimaxillary, or upper jaw CBCT scans between January 2017 and April of 2021 (*n* = 443). Using this population size, with a 5% margin of error and a 95% confidence interval, the power calculation using an online sample size calculator (http://www.raosoft.com/samplesize.html), indicated a minimum sample size requirement of 206 digital imaging and communications of medicine (DICOM) files for identifying significant differences in between-group comparisons. Nevertheless, to maintain estimates at an optimal level of precision against the effect of size reduction due to exclusions and dropouts, and considering a reported prevalence rate of mucosal thickenings at patient-level of approximately 35% [23], the study sample was increased by 72 DICOM files; thus, leading to a definite inclusion of 278 files for different assessments. The inclusion criteria were as follows: patients aged ≥ 18 years with both MS entirely visible on the CBCT scan and the absence of pathological conditions or upper jaw deformities. Conversely, the exclusion criteria applied were poor quality of CBCT images related to low-resolution, motion artifacts, and scattering/beam hardening artifacts, preventing the accurate recognition and delineation of anatomical structures; limited field of view that does not allow the visualization of the MS anatomy; neoplastic, traumatic, congenital, or pathological lesions of the upper jaw and/or MS; evidence of previous craniofacial or MS surgery; and ongoing orthodontic treatment. Given that MS increases in size until the end of the 18th year [30], patients younger than 18 years old were excluded from the scope of this study to avoid biases related to the growth and development of anatomical landmarks.

### Image acquisition and assessment

CBCT images included complete skull, bimaxillary, or maxillary scans were acquired with the i-CAT^®^ 17–19 system (Imaging Sciences International, Inc., Hatfield, Pennsylvania, USA) operating at 120 kVp, 18–48 mA, 26.9 s of exposure time, and 0.2 mm isotropic voxel size. The fields-of-view ranged from 16 × 6 cm to 23 × 17 cm depending on the image sizes. To avoid measurement bias related to head posture during scanning, CBCT images were taken with respect to the Frankfort horizontal and midfacial planes. The images were saved using i-CATVision 1.9^®^ software (Imaging Sciences International) and assessed synchronously by two Oral and Maxillofacial Surgery Senior Residents (C. B-N. and S. U-d.V.) previously trained and calibrated by two Maxillofacial Radiologists (C.I. S-N. and J.L O-C); thus, precluding a biased interpretation of tomographic parameters that might weaken the reliability of the results. The calibration was carried out using printed instructions and reference images illustrating examples of the possible morphological changes, mucosal thickenings, and morphometric measurements. All observations and measurements were achieved on both sides in each CBCT seen directly on a computer screen under appropriate light intensity and using the magnification tool to increase the image and the maximum intensity projection (MIP) mode for improving contrast. For linear measurements the analyses were done using the distance measuring tool of i-CATVision^®^ software, whereas for angular measurements the images were saved as JPEG files and processed using an image analyzer system (AxioVision 3.1^®^, Carl Zeiss^®^, Oberkochen, Germany). When discrepant measure data were established among the examiners, new evaluations were accomplished and any further disagreement was resolved by discussion until a consensus was reached.

### Tomographic data collection

**Fig. 1:**
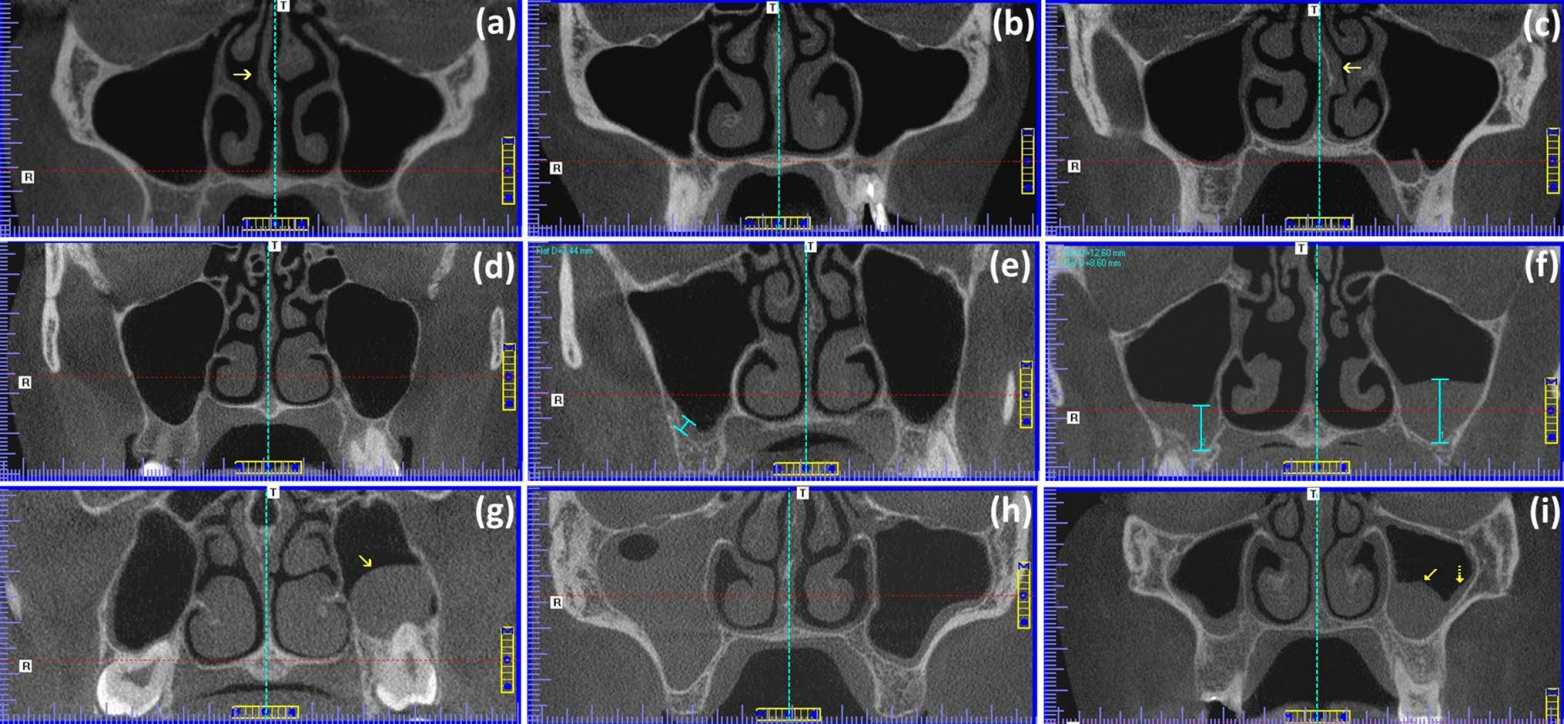
Representative CBCT images of MS with different mucosal membrane status. The lines were enhanced using the AxioVision^®^ software to illustrate the path by which the measurements were obtained. Pictures **a**–**c** show different characteristics of the nasal septum regarding to middle line as detected through coronal sections: **a** right septal deviation (yellow d arrow), **b** straight septum, and **c** left septal deviation (yellow arrow). Images **d**–**i** depict the appearance of the diverse mucosal thickenings: **d** absence of sinus membrane thickening bilaterally; **e** flat, shallow thickening ≥ 2 to 4 mm according to the measurement obtained from right MS; **f** bilateral flat, shallow thickening > 4 mm; **g** semispherical membrane thickening in the left MS (yellow arrow); **h**, almost complete occupation of the right MS; and **i**, mixed flat (yellow dotted arrow) and semispherical (yellow solid arrow) mucosal thickenings detected in the left MS (colour figure online)

**Fig. 2:**
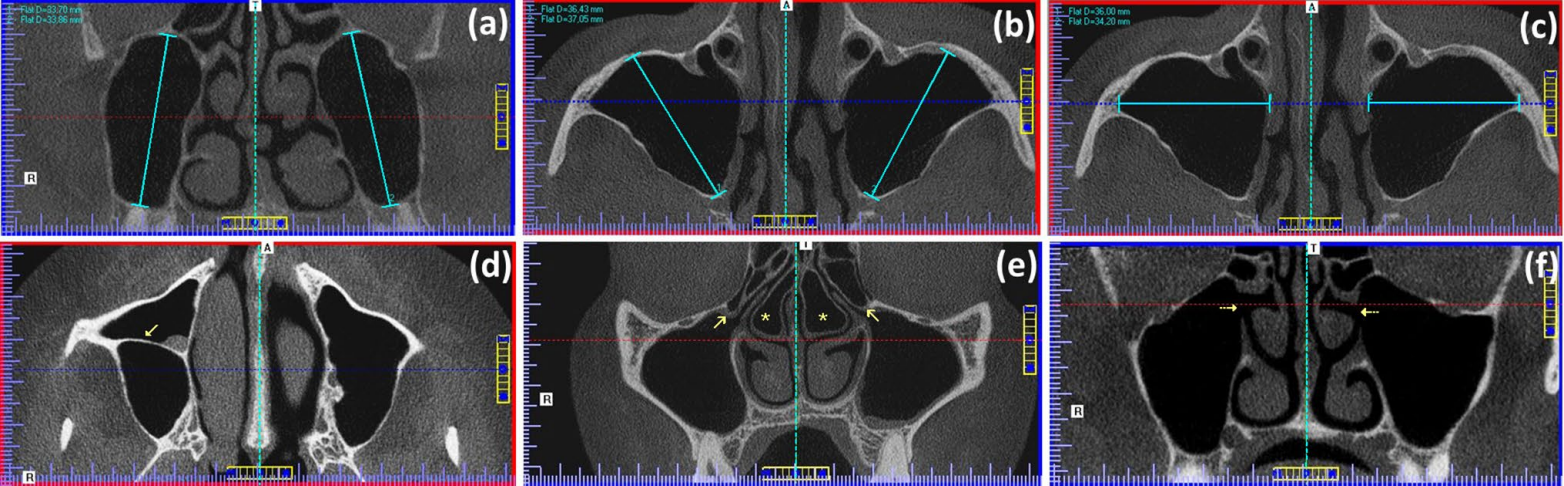
CBCT scans of MS and related anatomical structures. Pictures **a**–**c** show the way of the measurement of different dimensions after enhanced using the AxioVision^®^ software as follows: **a** maximal craniocaudal (height), **b** maximal anteroposterior (depth), and **c** maximal transversal (width) dimensions. Picture **d** represents an axial view demonstrating the presence of an antral septum (solid arrow). Image **e** depicts the anatomical location of the Haller cells (solid arrows) and concha bullosa (asterisks) as detected through coronal sections. Figure **f** corresponds to a coronal view demonstrating the presence of bilateral accessory maxillary ostia (dotted arrows)

**Fig. 3:**
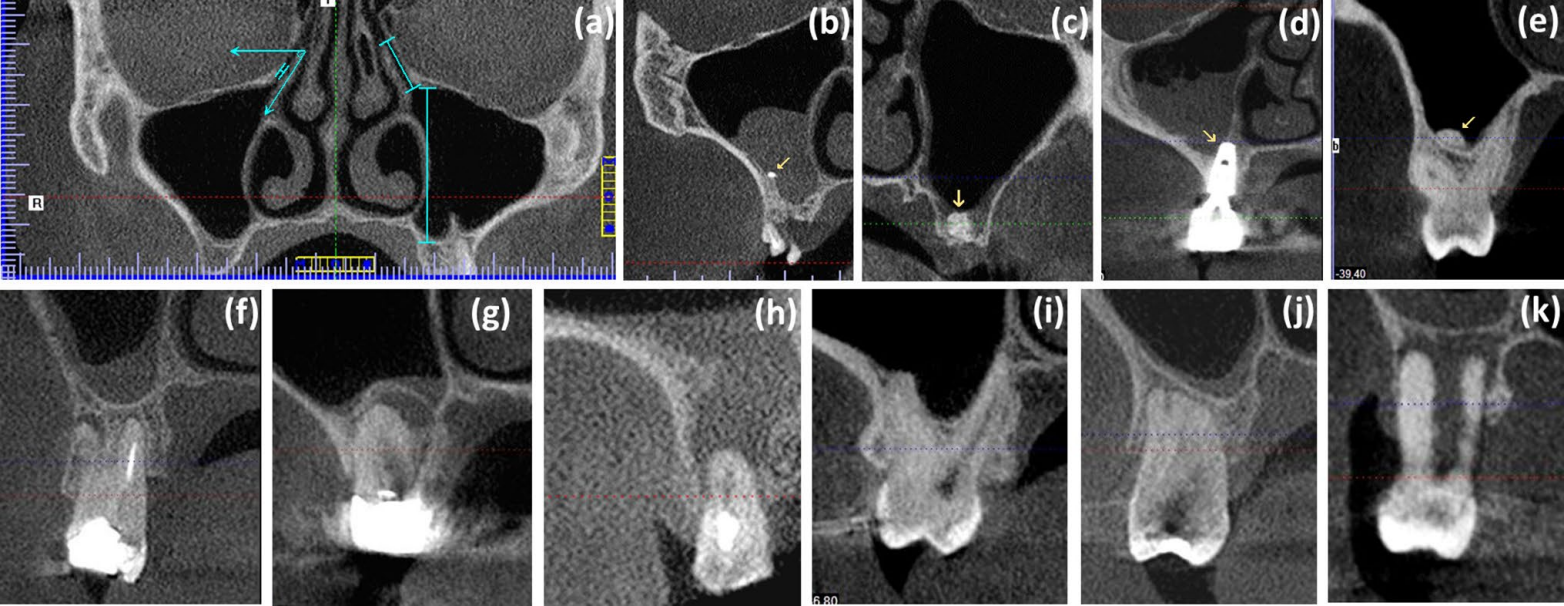
Typical CBCT images depicting **a** the way of measuring the angulation of the uncinate process with respect to the horizontal plane, as well as the height of the ostium, and the length and minimum width of the infundibulum. Additional pictures show mucosal thickenings associated to the presence of intrasinus foreign bodies consisting of **b** endodontic debris extrusion (yellow arrow), **c** graft material dislodged to the MS (yellow arrow), and **d** a dental implant penetrating slightly the sinus floor (yellow arrow). Image **e** shows a maxillary antrolith (yellow arrow) located on the sinus floor. The bottom row illustrates both the periapical and periodontal statuses as follows: **f** a gap is present between a periapical lesion and the sinus floor; **g** periapical lesion in contact with the sinus floor; **h** periapical lesion penetrating the sinus floor; **i** absence of periodontal bone loss; **j** slight-to-moderate periodontal bone loss; and **k** and severe periodontal bone loss (colour figure online)

Data collection was performed in several stages. In the first stage, the following patient-related parameters were recorded: gender (i.e., *male vs female*), age at the time of CBCT acquisition, the MS mucosal status (i.e., *healthy vs thickened*), type of sinus involvement (i.e., *unilateral vs bilateral*), and nasal septum deviation ≥ 4 mm from the midline (i.e., *none vs right/left deviation*) [46] (Fig. 1a–c). Mucosal thickenings were identified as non-corticated hyperdense straps running parallel to MS bony walls and considered abnormal if they showed thickness in at least one of the three orthogonal planes. The thickness of the mucosa was recorded based on the maximum perpendicular distance from mucosal surface to underlying bony plate and stratified according to the previous published criteria [10] with modifications as: (1) inconspicuous/no thickening of the sinus membrane; (2) flat, shallow thickening ≥ 2 to 4 mm; (3) flat, shallow thickening > 4 mm; (4) semispherical thickening of the membrane; (5) complete/almost complete occupation of the sinus; and (6) mixed flat and semispherical thickenings (Fig. 1d–i). Ostium obstruction secondary to mucosal thickening was further documented. Thereafter, using axial, coronal, sagittal, and panoramic reconstructions, the MS and their related anatomical structures were evaluated for the following features bilaterally as follows:

- The alveolar process was classified with reference to the presence/absence of teeth as *non-atrophic*, completely dentate *vs atrophic/partially atrophic*, with completely edentulous maxillary segments or with at least one missing tooth, excluding third molars [51].
- The maximum craniocaudal (height), anteroposterior (depth), and transversal (width) dimensions of MS were measured in order to calculate the volume of the antral cavity, based on the equation: $\mathrm{Vol}=\mathrm{height}\times \mathrm{depth}\times \mathrm{width}\times 0.5$, according to the previously defined criteria [25] (Fig. 2a–c). While the height of the MS was estimated on coronal images, as the distance among the highest point of the roof and the lowest point of the floor, the measurements the depth and width were performed on axial planes calculating the distances between the most anterior/posterior points and between the outermost/medial wall of the sinus, respectively.
- The *presence*/*absence* of antral septa, concha bullosa, Haller cells, and accessory maxillary ostia, were also recorded. Antral septa, described as walls of cortical bone within the MS [2], were diagnosed taking into account a minimal expansion of 2 mm in any of the orthogonal planes [22] (Fig. 2d). Concha bullosa was defined as the presence of pneumatization of any size on the left or right sides within the middle nasal turbinate [2, 46] (Fig. 2e). Haller cells were recognized as ethmoid air cells located along the medial orbital floor above the MS ostium and extending into the sinus [2, 33] (Fig. 2e). Accessory maxillary ostia were identified, using coronal views, as extra openings located on the anterior part of the posterior fontanelle of the lateral nasal wall [7, 48] (Fig. 2f).
- In addition, different quantitative variables were measured in the coronal view including (a) the angle between the horizontal plane and the vertical axis of the uncinate process (Fig. 3a) [26]; (b) the ostium height, measured from the roof the ostium to the lowest point of the corresponding MS (Fig. 3a); (c) the length of the infundibulum, measured as the distance between the center of the ostium and the uppermost point of the uncinate process (semilunar hiatus) passing through the infundibulum (Fig. 3a) [2]; (d) and the minimum infundibulum width, estimated as the smaller distance among the infundibulum walls (Fig. 3a).
- Alternatively, the assessment of iatrogenic variables related to surgical or dental treatments included the register of the *presence/absence* of foreign bodies such as endodontic debris (Fig. 3c) or guided tissue regeneration materials dislodged to the MS (Fig. 3c), and dental implants penetrating the sinus floor, (Fig. 3d). Likewise, the exploration of pathological variables was aimed at evaluating the *presence/absence* of antroliths (Figs. 3e) as well as periapical and periodontal health statuses. Periapical health was categorized based on the relationship of the periapical lesions and the sinus floor as *normal periapical structures* (i.e., absence of pathological hypodensities); or *presence of periapical lesion*, when there were periapical lesions present either separate from (Fig. 3f), in contact (Fig. 3g); or entering (Fig. 3h) the sinus floor [31]. In turn, periodontal status was assessed simultaneously in panoramic and coronal views and stratified according to the maximum degree of bone loss with reference to root length as *absent*, < 15% tomographic bone loss (Fig. 3i); or *slight-to-severe periodontal bone loss*, including cases with moderate breakdown (15–33%, Fig. 3j), or with severe bone defects (extending to mid-third of root and beyond, Fig. 3k) [47].

### Statistical methods

The analytical process was accomplished with a standard statistical program (SPSS, v.27.0, IBM, Armonk, NY) following three sequential steps. Initially, the intra-observer variability was estimated as the difference between two measurements of each variable performed by the same researchers with 20 DICOM files selected applying a simple random sampling process and using the Cohen’s kappa (*κ*) or the weighted kappa (*κ*_w_) statistics for categorical variables and the intraclass correlation coefficient (ICC) for quantitative variables. The time lag among test–retest was 12 months. The interpretation of results was based on the following definitions: values < 0.40 indicated poor reproducibility; 0.40–0.80 indicated fair to good reproducibility; and values > 0.80 indicated excellent reproducibility.

Thereafter, bivariate analyses were conducted in order to determine differences in the variables under study regarding MS mucosal status and to detect potential indicators for association with the mucosal thickenings. For continuous data, the mode of distribution was analyzed using the Kolmogorov–Smirnov test. Given that the variables were normally distributed, they were analyzed using the independent samples *t* test after homoscedasticity was confirmed using Levene's test. Additionally, Pearson’s correlation coefficient analysis (*r*) was used to evaluate the possible correlations between quantitative variables and Pearson’s chi-square test (*χ*^2^) was used for qualitative data.

Finally, univariate and multivariate binary logistic regression analyses were used to determine the strength and independence of association of significant candidate variables with the mucosal thickenings after adjusting for confounding variables with significance values < 0.20 identified in the bivariate comparisons. For this purpose, all quantitative data were dichotomized according to the optimal cut-offs points obtained from receiver operating characteristic (ROC) curve analysis. Positive associations occurred when the odds ratio (OR) was greater than one and the confidence interval (CI) did not include a value of 1 on any of the constructs. Furthermore, the Hosmer–Lemeshow test was applied to evaluate the goodness-of-fit of the logistic regression models. Also, pairwise interaction analyses were performed for those covariates that threw confounding associations. Statistical significance was set at a value of *P* < 0.05.

## Results

The study sample involved 278 CBCT DICOM files obtained from 114 males ranging in age from 18 to 74 years (mean 42.68 ± 17.00) and 164 females ranging in age from 18 to 86 years (mean 49.12 ± 17.23). The optimal cut-off point for age was set at 47.50 years. For each DICOM file, revealing both right and left MS (*n* = 556), a total of 21 variables were collected and assessed. The information obtained at patient-level revealed that 28.80% (80 patients) exhibited bilateral MS healthy mucosa and 71.20% (198 patients) had MS mucosal thickenings. Among these, 101 patients (51.00%) presented a unilateral thickening, while 97 (49.00%) presented bilateral thickenings. At the same time, the data gathered at sinus-level showed that 259 MS (46.60%) presented healthy mucosa, while 297 MS (53.40%) had a thickened mucosa. The most frequent mucosal thickening was the flat, shallow thickening ≥ 2 to 4 mm (33.70%), followed by the semispheric thickening of the membrane (23.60%), the flat, shallow thickening > 4 mm (22.90%), the mixed flat and semispheric thickenings (18.50%), and just a small number of cases revealed a complete occupation of the MS (1.30%). In general, the reproducibility analyses showed values ranging between 0.875 and 1.000, demonstrating an excellent intra-observer reliability (*P* < 0.001; ICC, Cohen’s *κ*, and *κ*_w_ tests) for all of the quantitative and categorical parameters tested. Complete ostium obstruction was detected in 31 MS (10.4%) with mucosal thickenings. Sinuses with > 4 mm of membrane thickening (including those with flat/shallow, semispheric, mixed flat and semispheric, and with complete occupation of the MS) were linked with a significantly greater proportion of MS with ostium obstruction than those with flat thickening ≥ 2 to 4 mm (13.2% *vs* 5.0%; *P* = 0.029, *χ*^2^ test, data not shown).

### Outcomes of bivariate comparisons

Bivariate comparisons of patient-related, anatomical, pathologic, and iatrogenic predictor parameters with reference to the MS mucosal status are outlined in Tables 1, 2, and 3. From Table 1 is apparent that although there were no significant differences (*P* > 0.05, *χ*^2^ test or unpaired *t* tests) between the MS mucosal statuses regarding any of patient-related variables, both gender and age had a confounding influence on the results, as they reached *P* values < 0.20. On the contrast, as can be seen from Table 2, those cases with thickened MS mucosa showed significantly greater mean values of maximum sinus and ostium heights, significantly lower of infundibulum length and width (all *P* < 0.05, unpaired *t* test), and a significantly higher proportion of atrophic/partially atrophic alveolar processes (*P* < 0.05, *χ*^2^ test) in comparison with those of the healthy MS mucosa. Moreover, while the maximum depth and the volume of MS also acted as confounders for the associations (*P* < 0.20, unpaired *t* test), the proportion of MS mucosal thickenings was statistically similar between the right and left MS *(P* > 0.05, *χ*^2^ test). It was also noteworthy that pathologic and iatrogenic predictors that contributed significantly (Table 3) to the outcome, were the foreign materials, the periapical status, and the tomographic bone loss (*P* < 0.05, *χ*^2^ test), while antroliths had a confounding effect. In addition, regardless of the MS mucosal status, except for infundibulum width and length, Pearson’s correlation analysis showed significant moderate-to-strong and positive correlations between all of the MS dimensions and ostium height (*r* = 0.477–0.866; all *P* < 0.001, data not shown); thus, indicating that the ostium height should increase synchronously with increasing of the height, depth, and width of the antral cavity.

**Table 1:** Summary of patient-level findings obtained from the study sample according to the MS mucosal status

Parameter	MS mucosal status		*P* value
	Healthy MS mucosa (*n* = 80)	Thickened MS mucosa (*n* = 198)	
Gender^a^			
Male	27 (33.80)	87 (43.90)	0.118^d^
Female	53 (66.30)	111 (56.10)	
Age (years)^b^	44.36 ± 18.64	47.33 ± 16.85	0.198^e^
Nasal septum deviation^a^			
Absent	29 (36.30)	80 (40.40)	0.521^d^
Present^c^	51 (63.70)	118 (59.60)	

^a^Data based on the number of individuals within each parameter according to the maxillary sinus mucosal status category

^b^Values are given as mean ± SD

^c^Including 86 cases (50.90%) with right nasal septum deviation and 83 cases (49.10%) with left nasal septum deviation

^d^Two-sided Pearson’s chi-square test (*χ*^2^)

^e^Two-sided unpaired *t* test

**Table 2:** Bivariate comparisons of anatomic variables according to MS mucosal status at sinus-level

Anatomic-related variables	MS mucosal status		*P* value
	Healthy MS mucosa (*n* = 259)	Thickened MS mucosa (*n* = 297)	
Maximum MS height (mm)^a^	35.75 ± 5.37	36.72 ± 5.71	0.038^c^
Maximum MS depth (mm)^a^	34.80 ± 3.57	35.37 ± 3.96	0.077^c^
Maximum MS width (mm)^a^	26.88 ± 4.87	26.99 ± 4.77	0.778^c^
MS volume (mL)^a^	17.35 ± 6.47	18.11 ± 6.48	0.168^c^
Alveolar process type^b^			
Non-atrophic	158 (61.00)	156 (52.50)	0.044^d^
Atrophic/partially atrophic	101 (39.00)	141 (47.50)	
Antral septa^b^			
Presence	117 (45.20)	127 (42.80)	0.567^d^
Absence	142 (54.80)	170 (57.20)	
Concha bullosa^b^			
Presence	96 (37.10)	110 (37.00)	0.994^e^
Absence	163 (62.90)	187 (63.00)	
Haller cells^b^			
Presence	108 (41.70)	110 (37.00)	0.261^d^
Absence	151 (58.30)	187 (63.00)	
Accessory maxillary ostium^b^			
Presence	84 (32.40)	89 (30.00)	0.531^d^
Absence	175 (67.60)	208 (70.00)	
Uncinate process angulation (degrees)^a^	51.37 ± 10.32	52.19 ± 10.39	0.352^c^
Ostium height (mm)^a^	31.66 ± 4.51	32.58 ± 4.64	0.019^c^
Infundibulum length (mm)^a^	10.54 ± 3.05	9.96 ± 3.01	0.026^c^
Minimum infundibulum width (mm)^a^	1.04 ± 0.47	0.95 ± 0.51	0.027^c^
Sinus side			
Right	128 (49.40)	150 (50.50)	0.799^d^
Left	131 (50.60)	147 (49.50)	

^a^Values are given as mean ± SD

^b^Values are given as *n* (%) of cases within each parameter according to the maxillary sinus mucosal status category

^c^Two-sided unpaired *t* test

^d^Two-sided Pearson’s chi-square test (*χ*^2^)

**Table 3:** Bivariate comparisons of pathologic and iatrogenic variables with reference to MS mucosal status at sinus-level

Pathologic and iatrogenic variables^a^	MS mucosal status		*P* value^f^
	Healthy MS mucosa (*n* = 259)	Thickened MS mucosa (*n* = 297)	
Foreign materials			
Presence^b^	5 (1.90)	16 (5.40)	0.033^e^
Absence	254 (98.10)	281 (94.60)	
Antroliths			
Presence	22 (8.50)	37 (12.50)	0.130^f^
Absence	237 (91.50)	260 (87.50)	
Periapical status^c^			
Normal periapical structures	215 (88.10)	213 (78.30)	0.003^f^
Presence of periapical lesion^d^	29 (11.90)	59 (21.70)	
Tomographic bone loss^c^			
Absent (< 15% bone loss)	171 (70.10)	149 (54.80)	< 0.001^f^
Slight-to-severe periodontal bone loss^e^	73 (29.90)	123 (45.20)	

^a^Values are given as *n* (%) of cases within each parameter according to the maxillary sinus mucosal status category

^b^Including 16 cases (76.20%) with dental implants penetrating the sinus floor, 2 cases (9.5%) showing foreign endodontic material within MS, and 3 cases (14.30%) with graft material dislodged to the MS

^c^Excluding 40 cases related to edentulous maxillary process

^d^Including 43 cases (8.30%) with space between periapical lesions and the sinus floor, 34 cases (6.60%) with periapical lesions in contact with the sinus floor, and 11 cases (2,10%) with periapical lesions entering the sinus floor

^e^Including 122 cases (23.60%) with slight-to-moderate (15–33%) tomographic bone loss and 74 cases (14.30%) with severe bone loss extending to mid-third of root and beyond

^f^Two-sided Pearson’s chi-square test (*χ*^2^)

### Results from binary logistic regression models

Information derived from univariate and multivariate binary logistic regression analyses of significant candidate variables for the association with MS mucosal thickenings before and after adjusting for the effects of those confounders identified in the bivariate analyses is depicted Table 4. Overall, the Hosmer–Lemeshow test probability values fluctuated from 0.052 to 0.392; thus, indicating an acceptable goodness-of-fit for each construct. It can be further appreciated that although the OR of mucosal thickenings was significantly increased (*P* < 0.05, Wald’s test) in the initial model for those cases with maximum MS height > 33.60 mm, atrophic/partially atrophic alveolar process category, and the presence of foreign materials, a confounding effect was apparent for these three covariates, as the association failed to reach statistical significance (*P* > 0.05) when adjusted for confounders individually. Conversely, there was no confounding of the association between the ostium height > 28.15 mm, infundibulum length ≤ 9.55 mm, minimum infundibulum width ≤ 0.50 mm, presence of periapical lesions, nor slight-to-severe periodontal bone loss, as all of these covariates, which were significant predictors for mucosal thickenings in the initial model, remained strongly and independently associated (*P* < 0.05) after adjusting for confounders. Further analyses performed between confounders revealed several pairwise biological interactions that might be involved in determining the MS mucosal thickenings (Table 5). This was evident between maximum MS height > 33.60 mm and maximum MS depth > 35.29 mm, as well as between the age stratum > 47.50 years regarding atrophic/partially atrophic alveolar process status and the presence of foreign materials within MS (all *P* values < 0.05).

**Table 4:** Summary of univariate and multivariate binary logistic regression analyses for the association of significant risk variables with MS mucosal thickenings before and after adjusting for gender, age, maximum MS depth, MS volume, and presence of antroliths

Clinical groups/parameters	Cases^a^		Univariate analysis		Multivariate binary logistic regression analysis		Calibration^d^
	Healthy MS mucosa	Thickened MS mucosa	Unadjusted OR (95% CI)^b^	*P* value^c^	Adjusted OR (95% CI)^b^	*P* value^c^	
Maximum MS height							
≤ 33.60 mm	97 (37.50)	81 (27.30)	Referent	0.011		0.060	0.237
> 33.60 mm	162 (62.50)	216 (72.70)	1.60 (1.12–2.29)		1.54 (0.98–2.42)		
Alveolar process type							
Non-atrophic	158 (61.00)	156 (52.50)	Referent	0.045		0.079	0.249
Atrophic/partially atrophic	101 (39.00)	141 (47.50)	1.41 (1.01–1.98)		1.40 (0.96–2.02)		
Ostium height							
≤ 28.15 mm	62 (23.90)	43 (14.50)	Referent	0.005		0.019	0.135
> 28.15 mm	197 (76.10)	254 (85.50)	1.86 (1.21–2.86)		1.74 (1.09–2.77)		
Infundibulum length							
> 9.55 mm	164 (63.30)	160 (53.90)	Referent	0.024		0.016	0.052
≤ 9.55 mm	95 (36.70)	137 (46.10)	1.48 (1.05–2.08)		1.54 (1.09–2.18)		
Minimum infundibulum width							
> 0.50 mm	235 (90.70)	245 (82.50)	Referent	0.005		0.005	0.083
≤ 0.50 mm	24 (9.30)	52 (17.50)	2.08 (1.24–3.48)		2.12 (1.25–3.59)		
Foreign materials							
Absence	254 (98.10)	281 (94.60)		0.041		0.061	0.179
Presence	5 (1.90)	16 (5.40)	2.89 (1.05–8.01)		2.67 (0.95–7.48)		
Periapical status^d^							
Normal periapical structures	215 (88.10)	213 (78.30)	Referent	0.004		0.005	0.249
Presence of periapical lesion	29 (11.90)	59 (21.70)	2.05 (1.27–3.33)		2.06 (1.25–3.40)		
Tomographic bone loss^d^							
Absent (< 15% bone loss)	171 (70.10)	149 (54.80)	Referent	< 0.001		< 0.001	0.392
Slight-to-severe periodontal bone loss	73 (29.90)	123 (45.20)	1.93 (1.34–2.78)		2.16 (1.42–3.29)		

^a^Values are given as *n* (%) of cases within each diagnostic group

^b^Odds ratio (95% confidence interval)

^c^Wald’s test

^d^Excluding data related to edentulous patients (*n* = 40)

^d^Hosmer & Lemeshow goodness-of-fit test

**Table 5:** Synergistic interactive effects among confounding variables with reference to the maxillary sinus mucosal thickenings

Biological interactions	OR (95% CI)^a^	*P* value^b^
Maximum MS height > 33.60 mm		
Maximum MS depth > 35.29 mm	1.46 (1.04–2.04)	0.029
Age stratum > 47.50 years		
Atrophic/partially atrophic alveolar process	1.57 (1.09–2.26)	0.014
Foreign materials	3.91 (1.10–13.86)	0.035

^a^Odds ratio (95% confidence interval)

^b^Wald test

## Discussion

MS is of paramount importance in diverse fields, including oral and maxillofacial surgery, otorhinolaryngology, head and neck surgery, as well in maxillofacial radiology. Therefore, CBCT images allow clinicians to diagnose various conditions that may be liaised with the generation and severity of sinus mucosal thickenings and that could affect the correct treatment planning. Notwithstanding, although there is overwhelming evidence that some patient-related [17, 23, 51, 52], anatomical [13, 17, 26], pathologic [3, 9, 15, 20, 32, 38, 41, 49], and iatrogenic [1, 32] variables are usual causes of mucosal thickenings, the information has been not only fragmentary, but also the results have been contradictory. At the knowledge of the authors, this is the first study intended to evaluate and compare comprehensively different risk variables associated with these mucosal abnormalities. Based on the reproducibility results observed within the study, it would possible to assume that this CBCT dataset constitutes reliable imaging findings for qualifying sinus alterations and their related variables with reliability and accuracy.

A better knowledge of the prevalence of MS mucosal thickenings and of the potential risk indicators related to its occurrence is of utmost importance for its prevention. In the current study, MS healthy mucosa was detected just in 28.80% of the patients, while 71.20% of them were diagnosed with mucosal thickenings. Based on the sinus-level data, healthy mucosa was detected in 46.60% and thickened mucosa in 53.40% of the MS. Although different prevalence rates of mucosal thickenings have been reported by several studies, in agreement to the former, other authors found close prevalence rates, with 60.60% of patients [42] and 53.60–66.00% of MS [29, 38, 42] presenting mucosal thickenings. However, lower prevalence rates have been also documented at both patient- [3, 23, 31, 39, 43] and sinus-level [3, 23, 31, 46]. As aforementioned, differences in the criteria used to define normal mucosal thickness might have had a great effect on prevalence estimates and may partly explain this variability [29, 43]. Although 2-mm was considered as a reliable threshold for pathologic mucosal thickening in this study, it has been acknowledged that some features related to the studied populations including ethnic diversity [3, 23, 27, 31, 46], indication of CBCT scans [3, 23], age groups [23, 27, 31, 46, 52], and differences in climate among geographical regions [38], may also influence the differences in prevalence rates. Furthermore, the diagnostic techniques used [27, 46] and dissimilarities in sampling criteria [38] may have led to great differences between the results across the studies.

Regarding the effect of patient-related variables, none of the potential explanatory variables analyzed in this study had a significant influence on the proportion of cases of thickened MS mucosa. In relation to gender and age, the findings have been conflicting as whereas some researchers associate male gender [3, 23, 38–40, 51] and aging [3, 23, 31, 39, 40, 52] with the thickened mucosal status, in agreeance with the current results, others have not found differences according gender [31, 52] or age [38, 51]. It was also noteworthy that although nasal septal deviation was found in 60.80% of the CBCT examinations, did not represent a risk indicator for the thickening of MS mucosa, instead it might actually be a regular anatomical variant of this structure, which is in coincidence with the data previously reported [8, 46], but contrasts with studies that have found an increased incidence and severity of MS disease related to increasing deviations of the nasal septum [12, 28]. Overall, these disagreeing results may be attributable to issues related to the inclusion/exclusion criteria which have a profound impact on the sample composition.

Another widely studied subject, for which divergent results have been described, is the influence of the anatomical variables on MS mucosal status. In this study, the ostium height as well as the infundibulum length and width were the foremost risk variables, as those cases with greater ostium height or lower infundibulum length and width were significantly linked with the proportion of MS mucosal thickenings in the univariate analyses and remained toughly and independently associated when adjusted for covariates. Altogether, these anatomical variables are important mainly due to the degree that they facilitate the sinus drainage to avoid the accumulation of secretions within the cavity and the establishment of a chronic inflammatory state. The present results partially parallel those reported by others who reported that some sinusopathies were significantly related to higher ostium height [17] and shorter infundibulum length [2]. Even so, it has also been described that the length of the infundibulum does not represent a parameter that influences the MS mucosal status [17]. These variations between studies could likely be attributable not only to differences in measurement techniques, but also to the severity and spectrum of mucosal thickenings [2, 17]. In addition, although relatively little has been published concerning the influence of infundibular width on the prevalence of MS mucosal thickenings, consistent with the data herein presented, a previous study based on computed tomography scans reported that the occurrence of a smaller infundibulum width is significantly associated with recurrent acute rhinosinusitis [5]. On the other hand, albeit in the bivariate and univariate analyses the maximum MS height > 33.60 mm and the atrophic/partially atrophic status of the alveolar process contributed significantly to the prevalence of mucosal thickenings, a confounding effect was evident after adjusting for other covariates in the final logistic regression model, as these parameters did not reach a significant association. Although these results partially coincide with those of other studies which indicate that the sinus size or volume [37] and the crest type [23, 51] do not have an independent relationship with the mucosal thickenings, since the confounding occurs when there are shared causes among the exposure and an outcome, it would be feasible to deduce that the heterogeneous effects of the male gender condition, aging, the increased depth and volume of MS, as well as the presence of antroliths, may be important modifiers for the impact of the increased MS height or the atrophic/partially atrophic status of the alveolar process for its association with thickened mucosal status.

The current study also analyzed a series of anatomical parameters that might be related to the deterioration of the mucociliary movement pattern and the obstruction of the ostium; thus, increasing the risk of sinus disease [26]. Accordingly, the proportion of cases showing antral septa, concha bullosa, Haller cells, and accessory maxillary ostia, as well as the uncinate process angulation were statistically similar between the study groups. Although there is great variability in the data obtained by other authors, the results obtained in this work indicated a close concordance with some published data concerning antral septa [2], concha bullosa [2, 28, 45], Haller cells [2, 32, 36], accessory maxillary ostium [50], and uncinate process angulation [26]. Nonetheless, discordant data have also been reported regarding Haller cells [4, 26] and the occurrence of accessory maxillary ostium [4, 7, 36, 44, 48]. It is important to point out here that the divergence between the outcomes might be attributable to factors related to anthropometric characteristics and the ethnic variability of the sample population, which may influence the anatomical configuration and location of these structures.

In regards to pathologic and iatrogenic variables, the presence of periapical lesions remained positively associated with the proportion of cases of mucosal thickening throughout the different analyses. In consistency with this finding, all the available studies [3, 18, 20, 27, 31, 41, 46] collectively indicate that periapical lesions might cause a precursor effect on the development of mucosal thickenings by means of passage of bacteria and bacterial by-products from periradicular tissues to the MS directly or through vascular anastomoses, lymphatics, and adjacent alveolar bone marrow spaces, thus involving the MS mucosa [31]. In addition to the above, alike in this work, several studies have highlighted that periodontal bone loss is associated with a greater prevalence of mucosal thickenings [3, 18, 20, 27, 31, 39]. In this context, it has been hypothesized that the possible mechanism for mucosal thickening is the presence of deep periodontal pockets which evoke a local reaction in the sinus mucosa, with edema, cell infiltration, fibrosis, or cystic degeneration in response to spreading of microorganisms and their products as well as tissue cytokines as occur for periapical pathosis [35]. However, other studies have reported contradictory findings [16, 24], so that the relation between the periodontal disease and the appearance of the sinus mucosa in CBCT images remains inconclusive [19]. This discrepancy between studies may be attributable to using different stratification categories for periodontal bone loss. This study further showed that the presence of foreign materials was also significantly related to the proportion of cases with mucosal thickening both in the bivariate and univariate analyses, which is in consonance to what was published in several case reports. Despite of the former, after adjustment for confounders, this association weakened considerably in the multivariate model, masking the role of foreign bodies on the occurrence of mucosal thickenings. Given that in order to draw clinically relevant conclusions, the results must be robust and consistent, that is, appear steadily in various comparisons, it does not seem appropriate to adhere to single significant differences [21]. Consequently, the clinical significance of foreign materials as risk variable for mucosal thickenings remains questionable and needs further investigation. Contrary to the aforesaid findings, though the presence of antroliths did not represent an indicator that influenced the mucosal status, it did have a confounding effect on the results. Although not much information is available about this relationship, this finding is in clear contrast with the previous researches, which suggests that the antroliths are usually accompanied by maxillary sinus inflammation involving mucosal thickening and fluid [14, 15]. While the reasons for the disagreement are not readily apparent, the evidence suggest that the antroliths can be formed, even in the absence of mucosal thickenings, as a consequence of the precipitation of calcium salts around a nidus or concentrated mucus, as observed in this study [15]; thus, confirming the confounding role of the antroliths on other covariates for association with mucosal thickenings.

One additional outstanding issue of the herein results was the detection of significant pairwise interactions between the maximum MS height > 33.60 mm and maximum MS depth > 35.29 mm, as well as between the age stratum > 47.50 years regarding atrophic/partially atrophic alveolar process status and the presence of foreign materials within MS for determining the MS mucosal thickenings. Taking into account that, alike in this study, the variations in the position of maxillary ostium have been positively correlated to MS height [11], and that aging may increase the prevalence of maxillary sinusitis [3] due to a more potential exposure to infections and inflammation [34, 52], it would be possible to assume that the increased MS height and aging can multiply the risk for mucosal thickening when the depth of the sinus is greater, there are foreign materials within the sinus cavity or tooth loss has occurred, thereby leading to impaired mucociliary clearance, mucus stasis, and MS mucosal disorders.

Finally, it is important to underline that in the current study several limitations were evident. First, this was a retrospective tomographic study and no clinical data regarding clinical sinus disease or symptoms were available. Hence, no relationships can be established between membrane thickening and the severity or the spectrum of maxillary sinus disorders. Further studies involving clinical examination in addition to the CBCT analysis are needed to validate the current results. In second instance, tomographic assessments were undertaken in a Colombian population which possesses a complex structure of individuals of different ethnic origins. This circumstance might compromise the generalizability of the study findings to other ethnic groups with different maxillary morphological features. Third, the current study also failed to evaluate the clinical characteristics related with the actual periodontal/periapical condition of the patients, so that the robustness of its association with the mucosal thickenings might have been underrated.

## Conclusions

While the increased ostium height, the decreased infundibulum length and width, the presence of periapical lesions and periodontal involvement might be the foremost indicator variables for MS mucosal thickenings, there are synergistic relationships among the increased sinus height and depth as well as aging regarding atrophic/partially atrophic alveolar process status and the presence of foreign materials that may be also associated with a greater proportion of these mucosal abnormalities.

## Data Availability

All data will be made available to the editorial board upon request.
